# Malaysian Health Literacy: Scorecard Performance from a National Survey

**DOI:** 10.3390/ijerph18115813

**Published:** 2021-05-28

**Authors:** Norrafizah Jaafar, Komathi Perialathan, Manimaran Krishnan, Nurashma Juatan, Masitah Ahmad, Teresa Yong Sui Mien, Kamarul Zaman Salleh, Affendi Isa, Suraiya Syed Mohamed, Nor Hanizah Abu Hanit, Wan Shakira Rodzlan Hasani, Emma Mirza Wati Mohamad, Mohammad Zabri Johari

**Affiliations:** 1 Institute for Health Behavioural Research, Ministry of Health Malaysia, Shah Alam 40170, Malaysia; norrafizah.j@moh.gov.my (N.J.); harini2678@gmail.com (K.P.); manimaran@moh.gov.my (M.K.); nurashmajuatan@gmail.com (N.J.); masitah.a@moh.gov.my (M.A.); teresa_yong@yahoo.co.uk (T.Y.S.M.); kamarulzaman.s@moh.gov.my (K.Z.S.); 2 Health Education Division, Ministry of Health Malaysia, Putrajaya 62590, Malaysia; affendi.isa@moh.gov.my (A.I.); suraiyacheah@yahoo.com (S.S.M.); 3 Institute of Public Health, Ministry of Health Malaysia, Shah Alam 40170, Malaysia; norhanizah.ah@moh.gov.my (N.H.A.H.); wshakira@moh.gov.my (W.S.R.H.); 4 Centre for Research in Media and Communication, Faculty of Social Sciences and Humanities, Universiti Kebangsaan Malaysia, Bangi 43600, Malaysia; emmamohamad@ukm.edu.my

**Keywords:** health literacy, healthcare, disease prevention, health promotion

## Abstract

Health literacy is an indicator of a society’s ability to make better health judgements for themselves and the people around them. This study investigated the prevalence of health literacy among Malaysian adults and provided an overall picture of the society’s current health literacy status, which has not been previously assessed. The study also highlighted socio-demographic markers of communities with limited health literacy that may warrant future intervention. A population-based self-administered survey using the Health Literacy Survey Malaysian Questionnaire18 (HLS-M-Q18) instrument was conducted as part of the National Health Morbidity Survey 2019 in Malaysia. The nationwide survey utilized a two-staged stratified random sampling method. A sample of 9478 individuals aged 18 and above, drawn from the living quarter list, participated in the study. The health literacy score was divided into three levels; limited, sufficient, and excellent. Findings showed a majority of the Malaysian population had a sufficient health literacy level in all three domains—healthcare, diseases prevention and health promotion (49.1%, 44.2%, and 47.5%, respectively)—albeit leaning towards the lower end of the category with an average score of 35.5. The limited health literacy groups were prevalent among respondents with older age (68%), lower education level (64.8%), and lower household income (49.5%). The overall health literacy status for Malaysia was categorized at a lower sufficiency level. Future health literacy improvements should focus on communities with a limited health literacy level to improve the overall score.

## 1. Introduction

The World Health Organization (WHO) defines health literacy as cognitive and social skills which determine individual motivation and the ability to gain access to, understand, and use information in ways that promote and maintain good health [1]. Studies have suggested that health literacy is an important contributor to existing health gaps [2,3,4]. An individual who is competent can access, understand, judge, and apply health information to healthcare, disease prevention, and health promotion; such individuals are considered to be health literate. Those who are health literate are able to facilitate their health decision making, such as by utilizing health care services optimally and practicing healthy lifestyles, to successfully apply the social determinants of health [5,6]. Those with low health literacy are associated with less participation in health-promoting and disease detection activities, propensity to make riskier health choices, weak management of chronic diseases, low adherence to medication, increased hospitalization and readmissions, and overall poor health outcomes which, in turn, result in increased morbidity and premature death [7].

A number of influencing factors may affect a person’s health literacy, such as education level, ethnicity, socioeconomic background, age, and disability. Studies have shown that people with lower socio-economic and educational backgrounds have poorer health awareness than those with from higher education and socio-economic levels [8]. Malaysia is a multi-ethnic, multilingual, and multi-cultural country with Malays (with the indigenous Bumiputras) being the predominant ethnic group, followed by Chinese, Indians, and other smaller ethnicities. Linguistically, Malaysian (the standardized version of the Malay language) is the official language of Malaysia [9]. It is also the primary language of knowledge and the medium of teaching and learning in the country [10]. This however, does not limit communication in other languages, which can be freely used by other ethnicities, such as Mandarin by Chinese and Tamil by Indians. In addition, English is the second official language and a compulsory subject taught at school [11].

The Malaysia population is predominantly concentrated in the 15–64 age group, which accounts for 69.5 percent of the total. The second largest cohort is the 0–14 age group (24.5 percent), and those aged 65 years or older make up 6.0 percent [12]. Despite the National Education Act gazette in 1961 [13], much of the population did not receive the national curriculum prior to the formalization of the National School Curriculum in the 1980s [14]; hence, differing levels of education were received by the population. As the population ages, care for the elderly needs special emphasis. Therefore, there is a need for continuous health literacy assessment to ensure the needs of all target groups are met.

In 2015, the first nationwide study among Malaysian adults for health literacy was conducted as part of the National Health and Morbidity Survey 2015 (NHMS) using a modified and translated version of the standard Newest Vital Sign (NVS) tool [15]. The functional HL tool is used to examines peoples’ understanding of information relevant to health for disease prevention [16]. The survey findings reported overall prevalence of adequate functional health literacy was only 6.6%, with the urban population reporting significantly higher adequate health literacy (7.8%) compared to the rural population (2.3%). The same study also reported adults with tertiary education were more adequately proficient in health literacy (11.0%) in comparison to those with primary education (2.4%) [15].

In NHMS 2019, a validated comprehensive HL tool known as Health Literacy Survey Malaysian Questionnaire18 (HLS-M-Q18) was used to address self-reported difficulties in tasks concerning decision making in health care (HC), disease prevention (DP), and health promotion (HP) [17]. The aim of this study was to assess the prevalence of Malaysian adult’s health literacy, which has not previously been investigated.

## 2. Materials and Methods

### 2.1. Recruitment and Sample Size and Sampling Determination

The NHMS 2019 is a nationwide survey covering both urban and rural areas in Malaysia. The survey targeted residences of non-institutional living quarters (LQs); institutional accommodation, such as hotel, hostels, and hospitals, were excluded. For the Health Literacy Module, all individuals aged 18 years and above living in Malaysia, residing in the living quarters (LQ) for at least 2 weeks prior to data collection were included. Institutional accommodation were excluded from the survey. Sample size was calculated using a single proportion formula for the estimation of prevalence. The sample size calculation was based on (a) variance of the proportion of the variable of interest (based on NHMS 2015 or other literatures); (b) margin of error; and (c) confidence interval of 95% [17].

The sample size was adjusted according to the need of the analysis, and the prevalence estimate was focused at national or state level. Based on the core objectives and the above-mentioned considerations, the optimum sample size required was 5676 LQs. The allocation of samples to states, and urban and rural locations, was conducted in proportion to the population size. A greater number of samples were allocated to states with larger populations, such as Selangor, Johor, and Sabah, and fewer samples were allocated to states with smaller populations, such as Melaka, Perlis, and Labuan. To ensure national representativeness, two-stage stratified random sampling was used. The two strata were the primary stratum, comprising states of Malaysia, including Federal Territories, and the secondary stratum, comprising urban and rural strata formed within the primary stratum. A total of 4703 living quarters (LQ) were successfully screened, resulting in a total of 10,472 respondents. Of these, 9478 individuals agreed to participate in the Health Literacy assessment, resulting in a 90.5% response rate [17].

### 2.2. Instrument

Data was collected nationwide between July and September 2019 using a self-administered HLS-M-Q18 questionnaire [18]. This questionnaire was adapted and compressed from the Health Literacy Survey European Questionnaire 47 (HLS-EU-Q47) [19], and was pretested in Selangor, Kuala Lumpur, and Sarawak using ratio-based sampling, which took into account population characteristics such as population size and ethnic density. For validation purposes, face and content validity were conducted among experts, researchers, stakeholders, and the technical team to ensure items in the questionnaire were valid for measurement of the specific domains. Instrument reliability showed all major domains in HLS-M-Q18 had a Cronbach’s Alpha value greater than 0.7 [18].

The questionnaire contained 18 items and the assessment focused on four dimensions of health literacy skills: ability to access, understand, appraise, and apply health information in three domains: health promotion, disease prevention, and healthcare. These were designed based on Likert-type responses (‘very easy’, ‘fairly easy’, ‘fairly difficult’, ‘very difficult’), with a final score awarded when respondents completed all 18 questions. All scores were transformed to a unified metric with a minimum score of 0 and a maximum score of 50, whereby 0 represents the ‘lowest possible’ and 50 represents the ‘highest possible’ health literacy score. The scores were divided into three levels; limited health literacy level (score 0–33), sufficient health literacy level (score > 33–42), and excellent health literacy level (score > 42–50) [6].

### 2.3. Data Collection Procedure

The HLS-M-Q18 was distributed together with other questionnaires under the NHMS survey. A self-administered approach requiring minimal guidance was used to obtain data from the selected respondents. The trained research assistants approached respondents, who were above 18 years old and able to read. Research assistants explained the objectives of the study, and informed respondents that their participation was voluntary and their anonymity was assured. After agreeing to participate, respondents provided written consent and the data collection sessions commenced. Respondents were given the options to answer via a tablet or through questionnaire handouts. Respondents were guided (in terms of questionnaire being read out, etc.) if they faced difficulties in reading and answering the questionnaire. The questionnaires were programmed into hand-held mobile devices for data collection [17].

### 2.4. Data Analysis

A descriptive weighted analysis using Statistical Package for the Social Sciences (SPSS) version 26 was used to measure the prevalence of health literacy within the Malaysian population. The prevalence of overall health literacy by socio-demographic subgroups was derived using complex sampling analysis, where locality was the main weight. Data normality was assessed and presentations of data are in the form of percentages, prevalence, and confidence intervals (CI). Data is presented by domains and overall results [17].

## 3. Results

### 3.1. Distribution of Health Literacy

Findings showed that a high number of Malaysians had good health literacy levels, with 40.7% (95% CI: 38.8, 42.5) possessing a sufficient level, 35.0% (95% CI: 33.0, 37.1) possessing a limited level, and 24.2% (95% CI: 22.6, 26.1) having an excellent level.

Table 1 shows that proportions of limited health literacy were higher among rural respondents (1.5%; 95% CI: 38.3, 44.9), have non-formal education (64.8%; 95% CI: 55.7, 72.9), earn less than RM1000 (42.5%; 95% CI: 44.0, 55.0), are male (37.2%; 95% CI: 34.3, 40.3), and are widowers or divorcees (48.1%; 95% CI: 43.1, 53.2).

The ratios of sufficient health literacy were higher among urban respondents (41.1%; 95% CI: 38.9, 43.3), in the age group of 40–44 years old (46.1%; 95% CI: 40.8, 51.5), who were Malay (44.0%; 95% CI: 42.3, 45.9), and had a tertiary education level (44.1%; 95% CI: 41.3, 47.1). Respondents with excellent health literacy level were similar to those of the sufficient health literacy level, and proportions were higher among the urban population (25.7%; 95% CI: 23.7, 27.9) with tertiary education level (31.2%; 95% CI: 28.1, 34.5), and a difference was seen in those with a higher income level of RM 10,000 and above (27.8%; 95% CI: 22.2, 34.2).

### 3.2. Health Literacy in the Domains of Health Care, Disease Prevention and Health Promotion

Health literacy domain analysis is shown in Table 2. Generally, the majority of respondents had sufficient health literacy level for all domains—health care [49.1% (95% CI: 47.2, 51.1)], disease prevention [44.2% (95% CI: 42.4, 46.1)], and health promotion [47.5% (95% CI: 45.7, 49.3)].

Among these domains, the limited health literacy group was most present [32.3% (95% CI: 30.4, 34.2)] in the disease prevention domain, whereas the sufficient health literacy group was most present [49.1% (95% CI: 47.2, 51.1)] in the health care domain and the excellent health literacy group was most present [25.9% (95% CI: 24.2, 27.6)] in the health promotion domain.

### 3.3. Health Literacy (HLS-M-Q18) per Items

As shown in Table 3, respondents reported that all 18 questions were relatively easy to comprehend in terms of finding and processing health information and services they received, and deciding the action to take next. Question Q3 of the health care domain was easiest to comprehend (58%), followed by question Q13 of the health promotion domain (55.7%).

## 4. Discussion

This Malaysian population-based survey revealed overall health literacy, and showed the majority of the population has sufficient and excellent health literacy, indicating that Malaysia’s health literacy is acceptable.

Regarding the whole spectrum of health literacy scores, the average score was 35.5 (out of the total score of 50). Although the overall population was within the acceptable/sufficient range (33–42) [19], the score fell at the lower end of the sufficient category.

The prevalence of Malaysian limited health literacy in the population is relatively similar to that of other countries, such as Ireland (40%), Germany (54.3%), Taiwan (34.4%), Sri Lanka (32.5%), and Vietnam (29.6%), for which comparable instruments were used [20,21,22,23]. A systematic review of 11 papers in Southeast Asia showed the overall prevalence of limited health literacy in South East Asia varies considerably within a range of 1.6%–99.5%, with a mean of 55.3% [24]. This relative pattern indicates the varying challenges faced by countries in improving health literacy.

There is also a possible association between sociodemographic characteristics and health literacy levels. Although not analyzed, prevalence data revealed limited health literacy was more prevalent amongst the older age group with a lower education level and lower household income. Sufficient or excellent health literacy levels were more evident in the population comprising the younger age group (under 50 years old), with higher education and higher income. These study results are consistent with those of other studies using similar HLS-EU-Q questionnaires, such as Canada [25,26], United States [27], Europe (Austria, Bulgaria, Germany, Greece, Ireland, the Netherlands, Poland, and Spain) [1], and Taiwan [28]. However, age and education were not associated with health literacy in Japan [29]. This could be due to the use of a Japanese Web survey study, which only included active Internet users and may not well represent the whole population.

In this study, the identified factors of older age, lower formal educational level, lower income, location, and unemployment status reflected social disparities and were interrelated with limited health literacy.

The link between older age and low health literacy is attributed to the decline in physical and cognitive capacities that may affect the understanding and judgement of this cohort [30].

A higher education level has been proven to be a significant determinant of a higher health literacy level. This is due to individual cognitive and social skills, which enable these individuals to gain access to and understand health information, and to use it to maintain their good health [31]. Similar results have been consistently reported in Iran [32], Germany [33], and Europe [1].

Other literature revealed that lower income is related to low health literacy [6]. Due to financial deprivation, lower education levels and lack of knowledge about to how to obtain information from sources other than healthcare providers [34], in turn equates to having less knowledge about medical conditions and treatment [3].

In contrast to rural communities, urban dwellers who possess sufficient health literacy tend to have better access to health care and better health information. The results also indicated rural individuals are likely to be limited in their attainment of higher education [35,36], live below the poverty line, account for a greater share of the population of older adults [37], do not have access to the Internet to seek information, and have less access to primary health care providers or specialists when seeking health care [38,39].

As reported in this study, female respondents have better sufficiency in health literacy than their male counterparts. This finding is comparable to those from studies conducted in United States [2], Saudi Arabia [40], Iran [32], and Korea [41]. Korean women have been shown to better understand medical or health information, and how to use this information in their daily lives, than men. Moreover, Korean women have higher educational levels than men, thus explaining their higher health literacy levels. Women who score higher in health literacy have also been found to be more able to locate health information and seek medical treatment for health issues, and indirectly have more medical interaction with health care providers, because they are more concerned regarding their health compared to males [42,43]. Nevertheless, no consistent patterns are evident between gender and health literacy because one of the sexes is more likely to have lower health literacy, as reported in Romania, Denmark [44], and Ghana [42].

Regarding marital status, it was reported that married individuals have sufficient and excellent health literacy compared to singles. Studies in Denmark [45] reported that individuals living with their families have higher health literacy. Compared to living alone, marriage fosters social interaction among family members through knowledge sharing, provision of support for making health decisions and communicating with healthcare professionals, and monetary support [2].

This study also demonstrated that students, followed by unpaid workers, homemakers, caregivers, and government employees, were among those who possessed the highest sufficient health literacy levels, whereas government employees led the excellent health literacy level group, followed by retirees and private employees. Government employees, particularly those with a minimum of higher education, were found to have a higher health literacy level than that of other occupations [46]. A study conducted in Iran [32] reported housewives or homemakers possessed higher literacy because they were more likely to be exposed to multimedia educational materials. Furthermore, higher literacy among women could be due to their role as caregivers, who provide care to sick members of the family, including children; hence, they tend to frequently engage with the health system when dealing with health issues [41].

Variations among Malaysian ethnicities, languages, and cultures also contribute to the disparity in health literacy levels [9]. Language differences among non-native ethnic groups can be a barrier to reading and understanding oral and written health information, and can further lead to low health literacy [47].

Retrospectively, it is worth highlighting that different instruments used to measure the health literacy level could result in different findings. For example, functional health literacy instruments would provide a more detailed indication of skills [48], whereas the instrument employed in this study resulted in a generic categorization of individual competencies. Conceptual health literacy covers a wide range of skills and competencies, which are developed throughout an individual’s lifetime, and relate to seeking, comprehending, evaluating, and using health information and concepts to make informed choices, reduce health risks, and increase quality of life [6].

To improve the health literacy performance of the overall population, it is recommended that creative and digital media are used to channel health information more widely and continuously, thereby enhancing the health literacy level of Malaysians as a whole. Guidelines for achieving these improvements should also be developed, in addition to regular monitoring and surveillance of population-based health literacy at the national level. These measures can significantly support decision making to improve population health literacy, and thus comprehensively contribute to further improvements of the population’s health.

## 5. Conclusions

Overall, the health literacy level among Malaysians can be categorized as sufficient, although it fell at the lower end of the sufficiency category. This current border-line situation of sufficient health literacy, in addition to the situation of limited health literacy, must be addressed by national health planners and policymakers involved with social determinants of health. The aim should be to develop appropriate public health and health promotion strategies and initiatives. Thus, the capabilities of those who are in the limited and sufficient literacy groups can be strengthened with the support and commitment of parties ranging from upper management to the community level.

## Figures and Tables

**Table 1:** Descriptive statistics of general health literacy level by socio-demographic characteristic.

Demographic Characteristics	Level of Health Literacy		
	Limited Health Literacy Level (Score 0–33)	Sufficient Health Literacy Level (Score > 33–42)	Excellent Health Literacy Level (Score > 42–50)
	Percentage (95% CI)		
Overall	35.0 (33.02, 37.11)	40.7 (38.89, 42.57)	24.3 (22.56, 26.02)
Residence			
Urban	33.2 (30.80, 35.70)	41.1 (38.87, 43.33)	25.7 (23.66, 27.89)
Rural	41.5 (38.29, 44.85)	39.4 (36.73, 42.20)	19.0 (16.87, 21.40)
Sex			
Male	37.2 (34.25, 40.28)	38.7 (35.99, 41.52)	24.1 (21.76, 26.54)
Female	32.7 (30.70, 34.85)	42.8 (40.81, 44.87)	24.4 (22.55, 26.42)
Marital Status			
Single	38.6 (34.86, 42.44)	39.4 (36.11, 42.80)	22.0 (19.12, 25.20)
Married	32.2 (29.95, 34.45)	42.0 (39.93, 44.15)	25.8 (23.72, 28.04)
Widow(er)/Divorcee	48.1 (43.06, 53.15)	33.5 (28.95, 38.32)	18.4 (14.85, 22.67)
Education Level			
No formal education	64.8 (55.71, 72.93)	26.2 (19.29, 34.48)	9.0 (4.89, 16.06)
Primary education	50.3 (45.79, 54.89)	35.9 (31.72, 40.32)	13.8 (11.34, 16.58)
Secondary education	32.4 (30.07, 34.78)	42.0 (39.70, 44.34)	25.6 (23.51, 27.84)
Tertiary education	24.6 (21.83, 27.67)	44.1 (41.25, 47.05)	31.2 (28.11, 34.54)
Unclassified	62.8 (34.40, 84.41)	28.3 (10.25, 57.59)	9.0 (02.29, 29.37)
Occupation			
Government employee	21.1 (17.73, 24.95)	42.9 (38.17, 47.71)	36.0 (31.17, 41.16)
Private employee	34.2 (30.71, 37.94)	41.1 (37.79, 44.55)	24.6 (21.95, 27.54)
Self employed	36.7 (32.85, 40.77)	39.8 (35.78, 43.95)	23.5 (19.69, 27.75)
Unpaid worker/Homemaker/caregiver	32.7 (29.43, 36.08)	44.0 (40.65, 47.34)	23.4 (20.21, 26.85)
Retiree	30.3 (23.70, 37.80)	40.3 (34.53, 46.37)	29.4 (23.74, 35.78)
Student	29.5 (21.69, 38.81)	49.3 (40.55, 58.17)	21.1 (15.54, 28.07)
Not working (unemployed, health problem, old age)	51.2 (46.45, 55.95)	32.0 (27.96, 36.23)	16.8 (13.96, 20.17)
Household Income Group			
Less than RM 1000	49.5 (44.04, 55.02)	36.8 (32.01, 41.87)	13.7 (10.35, 17.85)
RM 1000–RM 1999	39.4 (35.27, 43.66)	39.5 (35.36, 43.75)	21.1 (16.98, 26.00)
RM 2000–RM 3999	34.5 (31.35, 37.81)	40.2 (37.01, 43.53)	25.3 (22.60, 28.12)
RM 4000–RM 5999	29.0 (25.40, 32.90)	43.5 (39.62, 47.51)	27.5 (24.08, 31.13)
RM 6000–RM 7999	33.8 (28.35, 39.75)	39.9 (34.93, 45.12)	26.3 (21.59, 31.56)
RM 8000–RM 9999	24.4 (18.20, 31.93)	49.6 (41.78, 57.45)	26.0 (19.43, 33.81)
RM 10,000 and above	31.4 (22.77, 41.64)	40.8 (33.63, 48.31)	27.8 (22.20, 34.18)
Ethnic Group			
Malay (included Orang Asli)	30.6 (28.70, 32.61)	43.9 (42.14, 45.75)	25.4 (23.53, 27.44)
Chinese	36.6 (31.32, 42.23)	36.0 (31.45, 40.74)	27.4 (23.07, 32.28)
Indians	30.3 (24.43, 36.86)	36.7 (31.83, 41.79)	33.0 (27.76, 38.81)
Bumiputra Sabah	38.6 (32.81, 44.71)	41.3 (35.45, 47.46)	20.1 (15.62, 25.43)
Bumiputra Sarawak	41.9 (34.59, 49.63)	37.5 (31.99, 43.32)	20.6 (14.55, 28.30)
Others	51.2 (42.44, 59.90)	38.0 (29.57, 46.39)	11.2 (07.21, 16.96)
Age Group			
18–19	40.1 (32.14, 48.62)	39.2 (31.88, 46.92)	20.8 (14.87, 28.18)
20–24	37.3 (32.12, 42.73)	43.0 (37.61, 48.61)	19.7 (16.02, 24.01)
25–29	31.7 (27.27, 36.54)	41.2 (36.62, 45.93)	27.1 (22.88, 31.75)
30–34	32.0 (27.08, 37.35)	37.6 (33.28, 42.20)	30.4 (24.71, 36.68)
35–39	31.1 (26.85, 35.77)	43.6 (38.94, 48.44)	25.2 (21.53, 29.34)
40–44	25.6 (01.44, 30.28)	46.1 (40.76, 51.49)	28.3 (23.63, 33.51)
45–49	29.7 (25.40, 34.35)	43.6 (38.73, 48.51)	26.8 (22.44, 31.57)
50–54	34.9 (30.70, 39.37)	42.0 (37.48, 46.64)	23.1 (19.38, 27.28)
55–59	35.7 (30.98, 40.69)	41.7 (37.43, 46.13)	22.6 (18.59, 27.18)
60–64	41.2 (35.63, 47.01)	38.9 (33.76, 44.26)	19.9 (16.15, 24.31)
65–69	49.5 (42.58, 56.43)	33.0 (26.88, 39.70)	17.5 (13.09, 23.07)
70–74	51.2 (43.72, 58.71)	29.4 (23.60, 36.02)	19.3 (13.84, 26.32)
75 and above	68.0 (60.90, 74.31)	21.0 (15.69, 27.40)	11.1 (07.42, 16.21)
Household Income Group			
Bottom 40%	36.6 (34.04, 39.14)	40.9 (38.55, 43.36)	22.5 (20.41, 24.77)
Middle 40%	32.6 (29.27, 36.13)	40.1 (37.03, 43.25)	27.3 (24.27, 30.53)
Top 20%	30.7 (22.90, 39.86)	42.0 (35.28, 49.06)	27.3 (22.06, 33.13)

**Table 2:** Health literacy by domain.

Domain	Limited Health Literacy Level (Score 0–33)	Sufficient Health Literacy Level (Score > 33–42)	Excellent Health Literacy Level (Score > 42–50)
	Percentage (95% CI)		
Healthcare	27.9 (26.01, 29.89)	49.1 (47.22, 51.05)	23.0 (21.25, 24.76)
Disease Prevention	32.3 (30.39, 34.20)	44.2 (42.42, 46.06)	23.5 (21.84, 25.25)
Health Promotion	26.6 (24.76, 28.60)	47.5 (45.68, 49.26)	25.9 (24.23, 27.64)

**Table 3:** Frequency table for health literacy (HLS-M-Q18) items.

Domain	On a Scale from “Very Difficult” to “Very Easy”, How Easy Would You Say It is to:		Very Difficult (%)	Fairly Difficult (%)	Fairly Easy (%)	Very Easy (%)	Mean
Health Care	Q 1	…understand the medication guides that come with your medicine?	2.2	9.6	48.6	39.6	3.26
	Q 2	…understand what to do in a medical emergency?	4.2	21.5	50.2	24.2	2.94
	Q 3	…judge how information from your doctor applies to you?	1.9	10.1	58.0	29.9	3.16
	Q 4	…judge when you may need to get a second opinion from another doctor?	3.2	16.6	54.3	25.9	3.03
	Q 5	…call an ambulance in an emergency?	3.9	15.3	44.2	36.6	3.13
	Q 6	…follow instructions from your doctor or pharmacist?	1.3	5.9	50.3	42.5	3.34
Disease Prevention	Q 7	…find information on how to manage mental health problems like stress or depression?	4.6	21.9	47.8	25.6	2.94
	Q 8	…understand health warnings about behavior such as smoking, insufficient physical activity, unhealthy eating and drinking too much alcohol?	2.7	9.1	47.9	40.2	3.26
	Q 9	…find information about vaccinations/immunization and health screenings (such as breast exam, blood sugar test, blood pressure, cholesterol level) that you should have?	4.8	20.5	48.2	26.5	2.96
	Q 10	…understand why you need health screenings (such as breast exam, blood sugar test, blood pressure, cholesterol level)?	2.9	12.9	53.2	30.8	3.12
	Q 11	…judge which health screenings (such as breast exam, blood sugar test, blood pressure, cholesterol level) you should have? *(Appraise/Evaluate)*	2.8	16.9	53.4	26.9	3.04
	Q 12	…judge when you need to go to a doctor for a check-up?	1.9	11.3	52.9	34.0	3.19
Health Promotion	Q 13	…understand advice on health from family members or friends?	1.5	7.6	55.7	35.3	3.25
	Q 14	…understand information in the media (such as Internet, newspaper, magazines) on how to get healthier?	3.4	12.8	48.1	35.7	3.16
	Q 15	…judge how where you live (such as your community, neighborhood) affects your health and well-being?	2.2	11.8	55.1	31	3.15
	Q 16	…judge how your housing conditions help you to stay healthy	1.3	8.9	55.5	34.3	3.23
	Q 17	…make decisions to improve your health? *(Apply)*	1.5	10.1	55.4	33.1	3.2
	Q 18	…take part in activities that improve health and well-being in your community? *(Apply)*	3.5	18.4	46.4	25.9	2.99

## Data Availability

The dataset that support the findings of this article belongs to the National Health and Morbidity Survey 2019: Non-Communicable Diseases, Healthcare Demand and Health Literacy study. At present, the data are not publicly available but can be obtained from the authors upon reasonable request and with the permission from the Director General of Health, Malaysia.
